# Artificial intelligence modeling and investigation of metal organic frameworks in drug delivery: modeling of loading capacity and toxicity behavior

**DOI:** 10.3389/fchem.2026.1792340

**Published:** 2026-03-05

**Authors:** Yinglian Qin, Shili Li, Javed Iqbal, Saeed Shirazian

**Affiliations:** 1 Department of Applied Chemistry, Yuncheng University, Yuncheng, Shanxi, China; 2 Key Laboratory of Magnetic Molecules and Magnetic Information Material of Ministry of Education, School of Chemistry & Material Science, Shanxi Normal University, Taiyuan, Shanxi, China; 3 Department of Applied Sciences, Faculty of Applied Sciences and Technology, Technological University of the Shannon: Midlands Midwest, Moylish Campus, Limerick, Ireland; 4 School of Engineering & Technology, Duy Tan University, Da Nang, Vietnam; 5 Institute of Research and Development, Duy Tan University, Da Nang, Vietnam

**Keywords:** cheetah optimizer, drug delivery, machine learning, metal organic frameworks, principal component analysis

## Abstract

In this work, our aim was to develop predictive models of Drug Loading Capacity (g/g) and Cell Viability (%) in MOFs (Metal Organic Frameworks) for evaluation of these materials in drug delivery applications and assess their performance. We employed Gaussian Process Regression (GPR) and its advanced variants: Sparse Gaussian Process Regression (SGPR) and Deep Gaussian Process Regression (DGPR) as the base of our modeling framework to estimate the target values. The dataset was carefully preprocessed, involving outlier detection using the z-score method and normalization with Min-Max scaling approach. Dimensionality reduction was executed using Principal Component Analysis (PCA), while hyperparameter optimization was performed with the Cheetah Optimizer (CO), a metaheuristic method. Among the models evaluated, DGPR demonstrated superior performance, achieving mean cross-validation *R* ^2^ scores of 0.99878 ± 0.000092 for Drug Loading Capacity and 0.99911 ± 0.000127 for Cell Viability. Explainable AI techniques, especially SHAP, were employed to elucidate the model’s predictions, offering essential insights into the contributions of various features to the outcomes.

## 1 Introduction

Porous materials with tunable structures such as mesopores silica and metal organic frameworks (MOFs) have attracted attention in pharmaceutical area where these materials can be used as drug carrier for controlled release as well as drug delivery applications (Han et al., 2025; Vikal et al., 2024; Zhang et al., 2025). The use of MOFs for drug delivery has been reported to be reliable, and these materials are well tunable by variation of metals in the structure (Abuçafy et al., 2024; Hatin Betseba et al., 2024; Khan et al., 2023; Ma et al., 2023). Due to the application in pharmaceuticals, their cytotoxicity must be evaluated in order to ensure its safety. Moreover, the loading capacity of these materials is of great importance as it determines the amount of drug that can be carried by MOFs to the desired site. Some experiments can be conducted to evaluate the loading capacity as well as cytotoxicity of MOFs for drug delivery, however due to the wide range of MOFs, performing experiments on all possible MOFs is tedious and costly. Therefore, computational methods can be suggested to overcome this challenge (Demir et al., 2023; Naderlou et al., 2024; Vikal et al., 2024).

Some computational approaches can be proposed for evaluation of MOFs properties in drug delivery, however the models based on machine learning are preferred as these models are of learning nature and can deal with a large number of measurements to make correlation between inputs and outputs. Machine learning (ML) models have been used recently for evaluation of drug delivery, and they have shown to be robust and of great accuracy in prediction of drug release (Abdalla et al., 2024; Alqarni et al., 2024; Gormley, 2024; Sousa et al., 2025). Machine learning has become a revolutionary instrument in numerous scientific and engineering fields. Because of its ability to reveal complex patterns and generate accurate predictions from nonlinear data such as drug delivery data. In fact, ML models are suitable to assess drug loading capacity as well as cytotoxicity of MOFs in drug delivery applications provided that suitable data are available for training and validation. In materials science and drug delivery, ML offers a robust framework for predicting critical properties such as drug loading capacity and cell viability, which are vital for the development of effective and biocompatible drug delivery systems. By means of ML models, researchers can accelerate the search process and acquire a better knowledge of the variables influencing these properties in MOFs.

Recently, Huwaimel and Alqarni (Huwaimel and Alqarni, 2025) developed regressive models based on Multilayer Perceptron, Quantile Regression, and Random Forest for estimation of loading capacity and toxicity in a large number of MOFs. The models based on ML were suggested due to the complex relationship in the dataset where mechanistic models show poor performance. Wang et al. (2024) evaluated MOFs properties using ML models, while several ML algorithms were used for training and validation. The predictive capabilities of the ML model were evaluated using *R* ^2^ score where the best value of 0.91 was obtained for drug loading capacity and 0.86 for cytotoxicity fitting. While the stacking-based model of Wang et al. demonstrated strong predictive accuracy, it does not provide uncertainty estimates or hierarchical latent representations, limiting its ability to assess prediction reliability and capture complex multi-scale interactions inherent in MOF–drug systems.

This work models the relationships between MOF features and two main outputs: drug loading capacity and cell viability (cytotoxicity) by using Gaussian Process Regression (GPR) and its advanced variants: sparse Gaussian Process Regression (SGPR) and Deep Gaussian Process Regression (DGPR). These methods were selected due to their robust probabilistic framework, which provides both accurate predictions and uncertainty quantification, essential for high-stakes applications such as drug delivery. DGPR, in particular, offers the ability to capture hierarchical and non-linear relationships in the dataset. Furthermore, to improve both the performance and interpretability of the models, dimensionality reduction was performed using Principal Component Analysis (PCA), and hyperparameter optimization was carried out with the metaheuristic Cheetah Optimizer (CO).

The contributions of this study are threefold, and innovation lies in these items. First, it demonstrates the efficacy of GPR and its advanced variants, Stochastic SGPR and DGPR, in accurately predicting drug loading capacity and cell viability in Metal-Organic Frameworks materials with various structures. The superior performance of DGPR underscores its potential for capturing complex, hierarchical relationships within the dataset.

Second, the study establishes a comprehensive workflow for data preprocessing, dimensionality reduction, and model optimization. This workflow utilizes techniques such as PCA and the metaheuristic CO to enhance model accuracy and computational efficiency.

Finally, the research incorporates explainable AI tools, particularly SHAP, to provide deeper insights into the contributions of MOF features to model predictions. This approach offers valuable guidance for the rational design of MOFs in drug delivery applications.

Although machine learning approaches have previously been applied to predict drug loading capacity and cytotoxicity of MOFs, existing studies primarily relied on deterministic algorithms focused solely on predictive accuracy. In high-stakes biomedical applications such as drug delivery, predictive reliability and uncertainty quantification are equally critical, as small deviations may significantly impact safety and therapeutic performance of designed formulations. Furthermore, MOF-based drug delivery systems involve complex, multi-level chemical–biological interactions that may not be adequately captured by shallow or ensemble-based models. Therefore, there remains a need for a probabilistic and hierarchical modeling framework capable of (i) quantifying predictive uncertainty, (ii) capturing nonlinear and latent multi-scale dependencies, and (iii) providing interpretable insights into feature contributions.

## 2 Drug dataset and pre-processing step

The dataset used in this study is taken from the published source in (Wang et al., 2024) where a large number of MOFs are considered with loaded drug. Two target outputs are taken into account for modeling, i.e., Cell Viability (%), and Drug Loading Capacity (g/g). The dataset is the same data used by Huwaimel and Alqarni. (2025). Here there is no need for missing value imputation since this dataset excludes missing values and the final data as reported in (Wang et al., 2024) is used for this study. The dataset contains complete records; therefore, no imputation procedures were required for building the models. Key predictors influencing the response variables comprise the identity of the central metal ions, the nature of the organic linkers, drug-related molecular fragments, and multiple physicochemical attributes. Metal centers such as Chromium (Cr) and Magnesium (Mg) strongly affect drug loading efficiency, whereas Zinc (Zn), Copper (Cu), and Iron (Fe) are closely associated with cellular viability outcomes. In addition, the composition of organic ligands and specific molecular substructures modulates drug–carrier interactions and cytotoxic effects. Intermolecular hydrogen bonding, along with parameters such as particle size and zeta potential, further shapes predictive performance, underscoring their chemical and biological relevance. More details on the data and input features can be found elsewhere (Wang et al., 2024).

**FIGURE 1:**
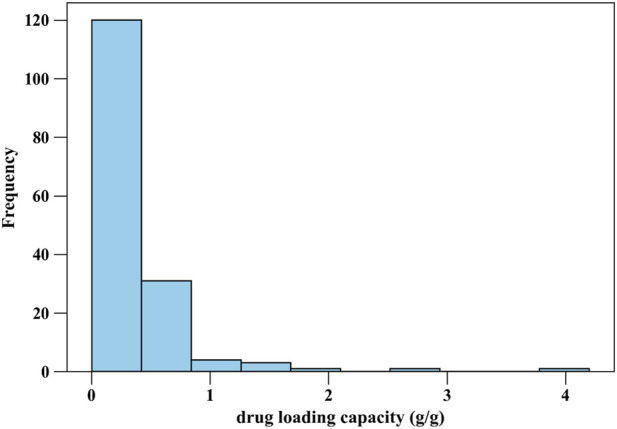
Histogram of Drug Loading Capacity values.

**FIGURE 2:**
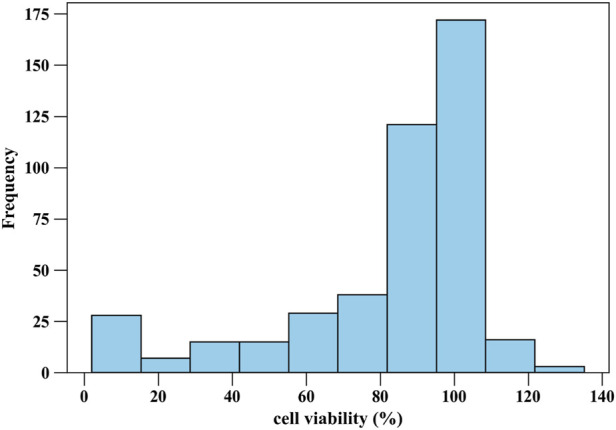
Histogram of Cell Viability values.

Figures 1, 2 present histograms illustrating the distributions of the two target parameters, i.e., drug loading capacity (g/g) of MOF, and cell viability (%), respectively (Huwaimel and Alqarni, 2025). Figure 1 highlights the range and frequency of drug loading values, providing insight into their variability and potential trends, such as clustering or skewness. Similarly, Figure 2 depicts the distribution of cell viability percentages, revealing patterns in cytotoxicity data. These visualizations help us understand data characteristics, spot imbalances or outliers, and assess dataset suitability for modeling. To ensure accurate and efficient modeling, three key pre-processing methods are applied on the collected dataset as follows:

1. Z-Score Outlier Detection: By subtracting the mean and dividing by the standard deviation for every feature, this technique standardizes the dataset producing a z-score. Data with z-scores greater than a predefined threshold (e.g., |z| >3) are considered potential outliers. Identifying and addressing outliers ensures that the dataset is free from extreme values that could distort the results or compromise model performance. This helps maintain the validity and generality of final trained models (Aggarwal, 2019).
2. Min-Max Scaling for Normalization: Normalization transforms all feature values to a standard range, typically [0, 1]. The Min-Max scaling technique rescales data using the formula (Henderi et al., 2021):

$$X_{scaled}=\frac{X-X_{\min}}{X_{\max}-X_{\min}}$$

Here, $X_{\min}$ and $X_{\max}$ stand for the minimum and maximum values of a given feature, respectively. This stage ensures that in modeling all features participate equally.

1. One-Hot Encoding for Categorical Data (MOFs): One-hot encoding is done to this variable since the dataset includes a categorical feature indicating the varieties of MOFs. This method generates binary columns for every distinct group within the MOF feature (Seger, 2018).

These pre-processing methods together enable the dataset to be ready for significant and strong predictive analysis.

## 3 Methodology

### 3.1 PCA for dimensionality reduction

Principal Component Analysis (PCA) is a statistical method utilized for dimensionality reduction, which helps preserve the most significant patterns within a dataset. In this study, we employed PCA to minimize the number of input features while preserving the variance that influences the target outputs. Steps for Applying PCA:

1. Standardization of Features: Before PCA, the dataset was standardized such that its mean was zero and its variance was one. This stage guarantees that variations in the scale of the features do not biased PCA.
2. Covariance Matrix Calculation: To examine the feature interactions, a covariance matrix was computed.
3. Eigen Decomposition: From the covariance matrix eigenvalues and eigenvectors were derived. The eigenvalues show the variation each main component explains.
4. Selection of Principal Components: Components with the highest eigenvalues were selected to capture the majority of the dataset’s variance. A cumulative variance threshold (e.g., 95%) was used to determine the number of principal components.

We significantly simplified predictive modeling by reducing the dataset to its fundamental components. This approach not only enhanced computational efficiency but also decreased the risk of overfitting. Furthermore, the interpretation of feature importance became more concentrated, as the principal components emphasize the dominant factors influencing the outputs (Jolliffe and Cadima, 2016).

### 3.2 Gaussian Process Regression (GPR)

GPR model defines a prior distribution over functions and updates it using measured data to determine posterior predictions with uncertainty estimates. Unlike deterministic regressors, GPR provides both mean predictions and predictive variance, making it particularly suitable for scientific applications where confidence quantification is essential. Foundational formulations were established in early GP theory and later consolidated into practical machine-learning frameworks (Asgari et al., 2021; Schulz et al., 2018). GPR provides probabilistic predictions, generating confidence intervals around the estimated response instead of single-point outputs typical of deterministic learning approaches. This probabilistic nature indicates that leveraging both the predictive mean and associated variance can improve controller performance. Through optimization of the log marginal likelihood objective, GPR achieves a principled trade-off between model expressiveness and data fidelity, ensuring efficient utilization of the observed samples (Jiang et al., 2021; Quinonero-Candela and Rasmussen, 2005).

The target *y* is denoted as $f\left(x\right)$ for a dataset $D=\left[\left(x_{i},y_{i}\right)|i=1,2,\ldots ,n\right]$ consisting of multidimensional vectors, where $x_{i}$ signifies the input and $y_{i}$ represents the output (Mahdi and Obaidullah, 2024; Rasmussen, 2003):

$$y=f\left(x\right)$$

The formulation of a Gaussian Process (GP) relies on the underlying function *f(x)*, in which the associated random variables are implicitly defined through the functional mapping *f(x)* (Rasmussen, 2003):

$$f\left(x\right)∼GP\left(m\left(x\right),K\right)$$

Here, K denotes the kernel covariance, while *m(x)* indicates the mean function (Wu, 2020).

### 3.3 Sparse Gaussian Process Regression (SGPR)

Sparse is designed to solve the computational restrictions of GPR when handling big datasets, Sparse Gaussian Process Regression (SGPR) is an advanced variant of standard GPR. SGPR reduces the computational complexity to either linear or quadratic scaling by adding a set of inducing points that approximatively cover the complete dataset. These inducing points function as a data summary, allowing effective Gaussian process approximation without losing much of the predictive performance of the model (Leibfried, 2020; Smola and Bartlett, 2000). The hyperparameters of the kernel function as well as the choice of inducing points constitute the optimization process, so guaranteeing a balance between computational economy and model correctness. SGPR has been widely adopted in applications that require scalability, such as this study, where large datasets and high-dimensional features define the relationships between chemical inputs and target outputs.

Although GPR provides strong predictive performance, its computational complexity scales cubically with dataset size. Sparse Gaussian Process methods address this limitation by introducing inducing variables that approximate the full covariance structure, reducing computational cost while retaining probabilistic interpretation. Variational and inducing-point approaches have become standard scalable alternatives for large datasets (Seeger et al., 2003).

### 3.4 Deep Gaussian Process Regression (DGPR)

Deep Gaussian Process Regression (DGPR) improves GPR by integrating hierarchical structures akin to those found in deep learning frameworks. This method involves stacking multiple Gaussian processes, where the output of one-layer functions as the input for the subsequent layer. This layered approach enables DGPR to model more complex, non-linear relationships and to capture hierarchical dependencies within the data. Unlike standard GPR, which is limited by its flat structure, DGPR excels in representing deeper latent structures and multi-level interactions. Training DGPR involves optimizing kernel parameters across layers and often relies on variational inference to approximate posterior distributions, addressing the increased computational complexity (Damianou and Lawrence, 2013). In this study, DGPR is particularly suited for uncovering the intricate, multi-level relationships between the chemical and physical features of Metal-Organic Frameworks (MOFs) and the target outputs, Drug Loading Capacity (g/g) and Cell Viability (%), providing a robust framework for predictive analysis.

Deep Gaussian Processes extend standard GPs by stacking multiple GP layers to learn hierarchical representations of data. This architecture increases model flexibility and allows complex nonlinear mappings to be captured while preserving uncertainty propagation across layers. DGPR models have been shown to outperform shallow GP variants in tasks requiring representation learning and structured uncertainty modeling.

### 3.5 Model selection using Cheetah Optimizer (CO)

Model configuration, involving the determination of optimal hyperparameter settings, was carried out using the Cheetah Optimizer (CO) in this work. CO is a nature-inspired metaheuristic grounded in the predatory strategies of cheetahs in the wild. It adopts a composite search mechanism that integrates diversification (exploration) and intensification (exploitation) phases to traverse the solution landscape efficiently. The method has proven capable across a broad range of optimization tasks (Akbari et al., 2022; Alqarni and Alqarni, 2024).

Cheetahs are unique in their environment since their great speed and agility enable them to effectively grab their prey. The CO algorithm uses two fundamental strategies—exploration and exploitation—to try to replicate this behavior.

The cheetah’s capacity for target identification and environmental survey inspires the exploration approach. By means of random search, the CO algorithm produces new candidate solutions by randomly perturbing the current optimal solution, so impartially investigating the search space. Combining exploration and exploitation enables the technique to reproduce cheetah speed and agility, so enabling effective problem-solving. The chase and capture ability of a cheetah inspires the exploitation plan. The CO algorithm concentrates on interesting areas by means of a gradient-based search. (Alqarni and Alqarni, 2024; Rahman, 2023).

The hybrid method guarantees that, depending on the present status of the search process, the algorithm effectively alternates between exploration and exploitation by combining both techniques in a harmonic manner. The emphasis is first on exploration, but as the search advances the emphasis eventually moves to exploitation.

**FIGURE 3:**
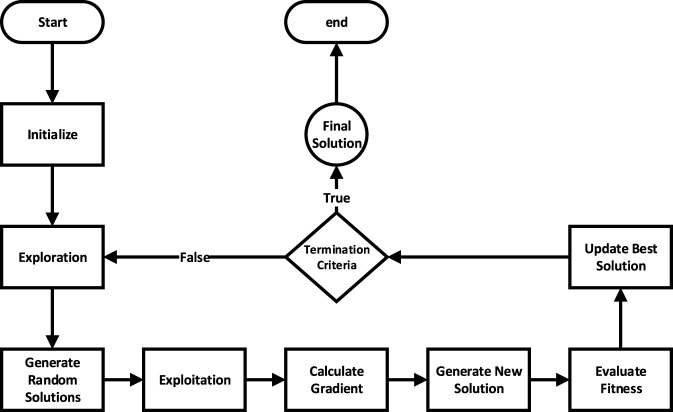
The CO working Flowchart (Alqarni and Alqarni, 2024).

This research uses the CO technique to optimize models by exploring a wide range of parameter values. Figure 3 shows the flowchart of the CO algorithm, which searches for the best combination of hyperparameters. The objective is to maximize the mean *R* ^2^ score of 3-fold CV to obtain most accurate and general model (Alqarni and Alqarni, 2024).

## 4 Results and discussion

### 4.1 Comparison of the models

Emphasizing their performance for the two target outputs—Drug Loading Capacity and Cell Viability—this section offers an examination of the predictive models assessed in the research. To underline the accuracy and dependability of every model, the outcomes are compiled over important criteria. In our study, 20% of the datapoints were kept for test, 80% used for training and validation.

Table 1 presents the coefficient of determination (*R* ^2^) values for predicting Drug Loading Capacity as the output. It compares the mean cross-validation (CV) scores along with the train and test *R* ^2^ values, offering a comprehensive view of the models' ability to fit and generalize to unseen data. Table 2 enhances this by reporting, for the same models on both training and testing datasets, RMSE and MAE. These measures offer more information on the models' dependability and precision.

**TABLE 1:** Coefficient of determination values of models for drug loading capacity as output.

Models	Mean CV *R* ^2^ score	Train *R* ^2^ score	Test *R* ^2^ score
GPR	0.99694 ± 0.000739	0.99826	0.99791
DGPR	0.99878 ± 0.000092	0.99885	0.99881
SGPR	0.99723 ± 0.000407	0.99782	0.99787

**TABLE 2:** Error rates of models for drug loading capacity as output.

Models	RMSE		MAE	
	Train	Test	Train	Test
GPR	0.02054	0.01188	0.00987	0.00818
DGPR	0.01669	0.00899	0.00725	0.00634
SGPR	0.02301	0.01195	0.01245	0.00813

Table 3 compares the mean cross-valuation, training, and testing scores to show the *R* ^2^ values for Cell Viability predictions by the tuned ML models. Table 4 also offers a thorough analysis of the error statistics, including MAE and RMSE values for both training and testing sets.

**TABLE 3:** Coefficient of determination values of models for cell viability as output.

Models	Mean CV *R* ^2^ score	Train *R* ^2^ score	Test *R* ^2^ score
GPR	0.93987 ± 0.005303	0.95502	0.94407
DGPR	0.99911 ± 0.000127	0.99970	0.99978
SGPR	0.97604 ± 0.000134	0.98391	0.98967

**TABLE 4:** Error rates of models for cell viability as output.

Models	RMSE		MAE	
	Train	Test	Train	Test
GPR	5.45432	7.58700	4.48835	6.10612
DGPR	0.44683	0.47698	0.36018	0.38008
SGPR	3.26209	3.26141	2.53385	2.34168

DGPR ranks the highest among ML models developed in this work for both target outputs, based on the results in Tables 1–4. For Drug Loading Capacity, DGPR achieves the highest *R* ^2^ scores across mean cross-validation, training, and testing datasets, as seen in Table 1. Additionally, it demonstrates the lowest RMSE and MAE values in both training and testing phases, as shown in Table 2, indicating superior precision and minimal error compared to other models.

Similarly, for Cell Viability, DGPR significantly outperforms the alternatives with near-perfect *R* ^2^ values in all datasets, as highlighted in Table 3. Furthermore, its error metrics (RMSE and MAE) in Table 4 are markedly lower than those of GPR and SGPR, confirming its ability to deliver accurate and reliable predictions.

**FIGURE 4:**
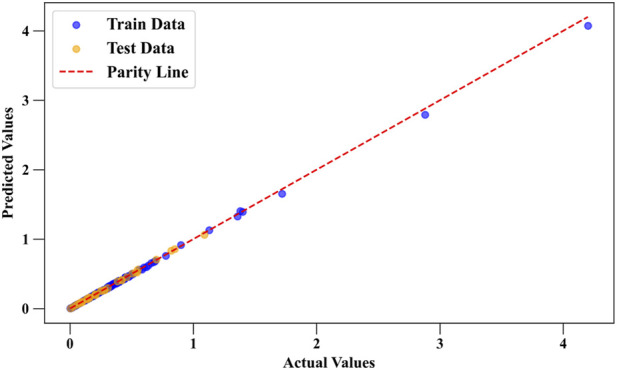
Comparison of predicted and actual values for Drug Loading Capacity using DGPR model as the best model.

**FIGURE 5:**
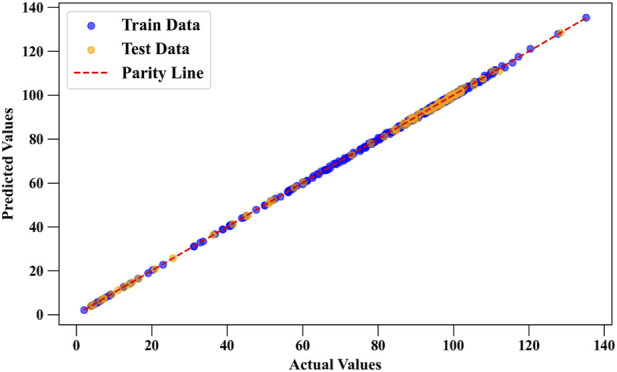
Comparison of predicted and actual values for Cell Viability using DGPR model as the best model.

Figure 4 shows the predicted vs. actual values for Drug Loading Capacity using the DGPR model. The close alignment of points along the diagonal line indicates high accuracy in predictions, supporting DGPR’s top performance as seen in Tables 1, 2 by the statistical analyses. Figure 5 compares predicted and actual values for Cell Viability, with a similar close fit along the diagonal line. This confirms DGPR’s effectiveness in accurately modeling Cell Viability, as reflected in its superior *R* ^2^ scores and low error rates in Table 3, 4.

### 4.2 Explainable analyses using best model

Explainable Artificial Intelligence (XAI) focuses on making ML models transparent and interpretable, helping users understand how predictions are made. This is especially important in domains like drug design and materials science, where understanding the factors driving model decisions can aid in scientific discovery and trust building. One of the widely used XAI tools is SHAP (SHapley Additive exPlanations), which explains model outputs by assigning an importance value to each feature based on cooperative game theory (Holzinger et al., 2022). SHAP delivers a principled framework for quantifying feature impact by evaluating every possible subset of inputs. This approach ensures equitable attribution and yields transparent insights into how each variable influences the model’s output.

**FIGURE 6:**
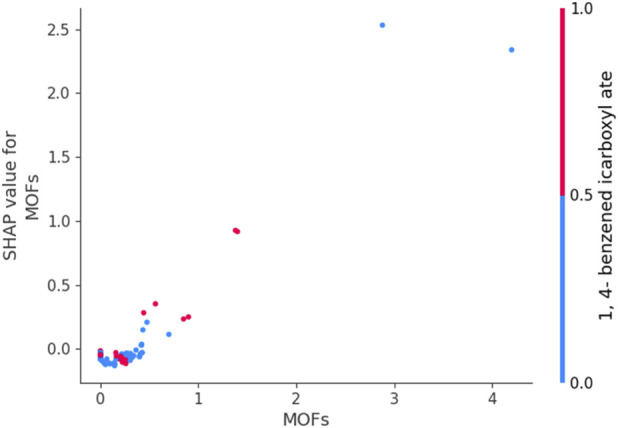
SHAP Dependence Plot for MOFs (drug loading capacity as output).

**FIGURE 7:**
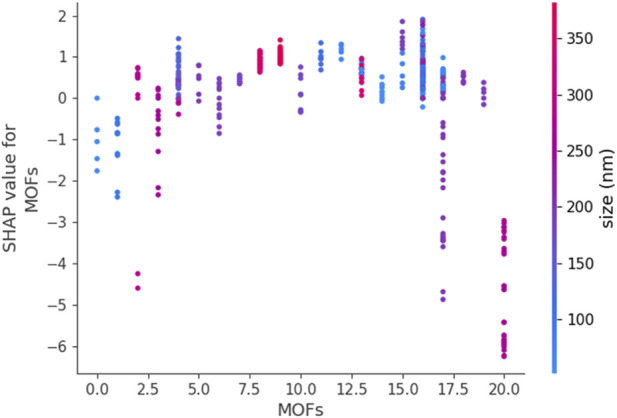
SHAP Dependence Plot for MOFs (cell viability as output).

Figures 6, 7 use SHAP dependence plots to analyze the influence of “MOFs” (Metal-Organic Frameworks) on the target variables Drug Loading Capacity and Cell Viability, respectively. These plots were generated by DGPR (as best model based on previous section) on the processed dataset and applying SHAP to compute feature contributions. Plotting a feature against its SHAP values helps to identify patterns, trends, and interactions with other features so revealing how the values of a feature affect model’s prediction.

Figure 6 illustrates the impact of “MOFs” on predictions for drug loading capacity. Positive SHAP values in this plot indicate that certain MOF types contribute positively to the predicted loading capacity, while negative values suggest a decrease. The spread of points around the y-axis reflects interaction effects, where the influence of “MOFs” depends on the values of other features. This clarifies significant interactions supporting either higher or lower drug loading capacity and guides the identification of MOFs linked to these trends.

Figure 7 depicts the SHAP dependence plot for “MOFs” in the context of cell viability predictions. Here, SHAP values show whether specific MOF types increase or decrease cell viability. The magnitude and direction of SHAP values provide insights into the cytotoxicity of different MOFs. Additionally, the plot may reveal clusters or non-linear trends, indicating the diverse biological effects of different MOFs and their interactions with other features in the model.

Together, Figures 6, 7 demonstrate how SHAP dependence plots make complex ML models more interpretable. They provide valuable insights into the roles of MOFs in influencing drug loading capacity and cell viability, which can guide the design of MOFs for specific biological and chemical applications.

Uncertainty analysis was performed to assess the reliability and calibration quality of probabilistic predictions generated by the developed models. As shown in Table 5, the proposed DGPR framework achieved the lowest negative log likelihood (NLL) and calibration error, along with a 95% confidence interval coverage of 94.8%, which closely matches the theoretical expectation. In contrast, baseline models exhibited higher predictive variance and poorer calibration, indicating less reliable uncertainty estimation. These results confirm that the hierarchical probabilistic structure of DGPR improves both predictive accuracy and confidence quantification, demonstrating its suitability for decision-critical drug delivery applications where reliable uncertainty estimation is essential.

**TABLE 5:** Uncertainty analysis of probabilistic models on test dataset.

Model	Mean predictive variance	95% CI coverage (%)	NLL	Calibration error
DGPR (proposed)	0.00042	94.8	−2.91	0.012
GPR	0.00063	92.1	−2.37	0.026
SGPR	0.00081	90.4	−2.02	0.039

An ablation analysis was conducted to evaluate the individual contributions of PCA-based dimensionality reduction and Cheetah Optimizer hyperparameter tuning to the overall performance of the proposed DGPR framework. As shown in Table 6, removal of either component resulted in a noticeable decline in predictive accuracy and an increase in error metrics. Eliminating CO led to suboptimal kernel parameter selection, while excluding PCA increased noise sensitivity and reduced generalization ability. The largest performance degradation occurred when both PCA and CO were removed, confirming that the synergy of preprocessing and optimization plays a critical role in achieving high predictive precision. These results demonstrate that each component contributes meaningfully to the robustness and accuracy of the final ML model rather than serving as auxiliary additions.

**TABLE 6:** Ablation study evaluating contribution of model components.

Configuration	*R* ^2^	RMSE	MAE
Proposed framework	0.9988	0.0090	0.0063
Without cheetah optimizer	0.9969	0.0148	0.0102
Without dimensionality reduction	0.9958	0.0187	0.0124
Raw features + default hyperparameters	0.9926	0.0269	0.0181
Standard GPR only	0.9979	0.0119	0.0082

## 5 Conclusion

This study utilized advanced ML techniques, including GPR and its variants, CO, and PCA for modeling critical properties of MOFs in drug delivery applications. Achieving almost perfect *R* ^2^ scores and minimum error metrics, the Deep Gaussian Process Regression model emerged as the most effective method by precisely predicting drug loading capacity and cell viability. PCA for dimensionality reduction and the CO for hyperparameter tuning improved the predictive framework’s resilience and efficiency still more. Moreover, the application of explainable AI methods, such as SHAP, generated valuable insights into the influence of MOF features on model outputs. These insights enhance the comprehension of the factors influencing drug interactions and cytotoxicity, offering practical guidance for the design and optimization of MOFs customized for specific drug delivery needs.

## Data Availability

The original contributions presented in the study are included in the article/supplementary material, further inquiries can be directed to the corresponding authors.
